# Downregulated ZNF132 predicts unfavorable outcomes in breast Cancer via Hypermethylation modification

**DOI:** 10.1186/s12885-021-08112-z

**Published:** 2021-04-07

**Authors:** Zhao Liu, Jiaxin Liu, Ruimiao Liu, Man Xue, Weifan Zhang, Xinhui Zhao, Jiang Zhu, Peng Xia

**Affiliations:** 1 Department of Surgical Oncology, The First Affiliated Hospital of Xi’an Jiaotong University College of Medicine, Xi’an, 710061 Shaanxi China; 2 Department of Gerontological Surgery, The First Affiliated Hospital of Xi’an Jiaotong University College of Medicine, Xi’an, 710061 Shaanxi China; 3 Department of Clinical Laboratory, Peoples Hospital of Xi’an (Fourth Hospital of Xi’an), Xi’an, 710004 Shaanxi China; 4 Department of General Surgery, Tongchuan Mining Bureau Central Hospital, Tongchuan, 727000 Shaanxi China; 5 Department of Breast Disease, Shaanxi Provincial Cancer Hospital, Xi’an, 710061 Shaanxi China

**Keywords:** ZNF132, Breast cancer, Bioinformatic, Methylation, Diagnosis, Prognosis

## Abstract

**Background:**

An important mechanism that promoter methylation-mediated gene silencing for gene inactivation is identified in human tumorigenesis. Methylated genes have been found in breast cancer (BC) and beneficial biomarkers for early diagnosis. Prognostic assessment of breast cancer remain little known. Zinc finger protein 132 (ZNF132) is downregulated by promoter methylation in prostate cancer and esophageal squamous cell carcinoma. However, no study provides information on the status of ZNF132, analyzes diagnosis and prognostic significance of ZNF132 in BC.

**Methods:**

In the present study, the expression of ZNF132 mRNA and protein level was determined based on the Cancer Genome Atlas (TCGA) RNA-Seq database and clinical samples analysis and multiple cancer cell lines verification. P rognostic significance of ZNF132 in BC was assessed using the Kaplan-Meier plotter. Molecular mechanisms exploration of ZNF132 in BC was performed using the multiple bioinformatic tools. Hypermethylated status of ZNF132 in BC cell lines was confirmed via Methylation specific polymerase chain reaction (MSP) analysis.

**Results:**

The expression of ZNF132 both the mRNA and protein levels was downregulated in BC tissues. These results were obtained based on TCGA database and clinical sample analysis. Survival analysis from the Kaplan-Meier plotter revealed that the lower level of ZNF132 was associated with a shorter Relapse Free Survival (RFS) time. Receiver operating characteristic curve (ROC) of 0.887 confirmed ZNF132 had powerful sensitivity and specificity to distinguish between BC and adjacent normal tissues. Bioinformatic analysis showed that 6% ((58/960)) alterations of ZNF132 were identified from cBioPortal. ZNF132 participated in multiple biological pathways based on the Gene Set Enrichment Analysis (GSEA) database including the regulation of cell cycle and glycolysis. Finally, MSP analysis demonstrated that ZNF132 was hypermethylated in a panel of breast cancer cell lines and 5-aza-2′-deoxycytidine (5-Aza-dC) treatment restored ZNF132 expression in partial cell lines.

**Conclusions:**

Results revealed that hypermethylation of ZNF132 contributed to its downregulated expression and could be identified as a new diagnostic and prognostic marker in BC.

**Supplementary Information:**

The online version contains supplementary material available at 10.1186/s12885-021-08112-z.

## Background

Breast cancer (BC) is one of the most frequent malignant tumors in females and the fifth leading cause of cancer-associated mortality in worldwide [1, 2]. Advancement in early detection and treatment has improved 5 year-survival rates of BC patients [3–8]. In 2018, approximately 62% of patients with no distant metastasis were diagnosed who displayed a favorable 5-year survival rate of 99% [9]. However, the 5-year survival rate of the patients with distant metastases decreases to 23% [10]. Therefore, early diagnosis and treatment improves survival time for BC patients.

Multiple mechanisms have been reported for breast carcinogenesis. These multiple mechanisms include: overactivation of oncogenes, overexpression of growth factors and receptors, and the silence of tumor suppressor [11–13]. Studies have confirmed that epigenetic alterations are involved in tumor progression [12, 14]. Aberrant DNA methylation of CpG islands is considered to be a vital mechanism to silence anti-tumor genes [15, 16]. Promoter methylation is an important marker of tumor cells. This is due to its role in tumor cell differentiation, proliferationand survival [17, 18]. Studies have identified a broad range of genes silenced by DNA hypermethylation in carcinogenic process [19]. However, aberrant DNA methylation inactivation of genes related tumor occurrence and development has not been well studied in BC. Zinc finger proteins are an important member of transcription factor family which play a vital role in human diseases including cancers [20]. Majority of zinc finger proteins contain Kruppel associated box (KRAB) domains. KRAB domains induce inhibition of the transcription of downstream genes [21]. Zinc Finger Protein 132 (ZNF132) is located at chromosome 19q13.4 and belongs to the zinc finger protein family. Low expression of ZNF132 has been reported in cancer and is associated with the cell growth, migration and invasiona ablity in prostate cancer and esophageal squamous cell carcinoma up to date. However, its role in BC remains unknown.

The study investigated the expression and clinical significance of ZNF132 based on the TCGA database and clinical samples analysis. Moreover, further exploration was performed to assess the status of ZNF132 in BC using multiple bioinformatics tools. The epigenetic alteration of ZNF132 was associated with its downregulated expression. Collectively, study findings demonstrated ZNF132 could be a new potential prognostic factor for BC and may serve as a promising therapeutic target for BC patients.

## Methods

### Analysis of the mRNA expression of the ZNF132 in multiple platforms

The mRNA expression of ZNF132 was explored among the tumor and normal tissues in the BC data of TCGA (http://cancergenome.nih.gov/) [22]. Meanwhile, ZNF132 expression and clinical outcome datas were also obtained from the Oncomine™ database (www.oncomine.org) [23] and UALCAN platform (http://ualcan.path. uab.edu/) [24]. Furthermore, the mRNA expression of ZNF132, along with other clinic-pathological data, was downloaded from the Xena platform (https://xenabrowser.net/).

### Clinical samples

Nineteen pairs of clinical samples including BC and normal adjacent tissues were collected from the First Affiliated Hospital of Xi’an Jiaotong University from January 2019 to March 2019. These patients did not receive any therapeutic intervention and signed an informed consent before surgery. All patients were finally histologically diagnosed by two pathologists based on World Health Organization (WHO) criteria. Ethical approval was provided by the First Affiliated Hospital of Xi’an Jiaotong University Ethics committee.

### RNA extraction and quantitative RT-PCR

RNA was extracted according to a previous protocol [25]. qRT-PCR was performed using the Bio-Rad CFX Manager detection system to assess the mRNA expression of ZNF132 between BC and normal tissues. The SYBR protocol followed the parameters: 95 °C for 30 s, 38 cycles of 5 s at 95 °C and 30 s at 55 °C. The primers used are as follow: ZNF132: forward: 5′-CCACAGTGTGATGCTGGAAAA CC-3′, reverse: 5′-GCTTTCTTGGTGGAAGGATCTGC-3′ and 18 s rRNA: forward: 5′-CGCCGCTAGAGGTGAAATTC-3′, reverse: 5′-CTTTCGCTCTGGTCCGTCTT-3′. The mRNA expression of the ZNF132 was normalized to 18S rRNA cDNA.

### Immunohistochemistry (IHC)

BC tissues were fixed in 4% formaldehyde at room temperature for 48 h in preparation. The ZNF132 antibody (BIOSS, Beijing, China; cat. no. bs-7150R, 1:200 dilution) was used for IHC detection. After incubation overnight at 4 °C, the analysis of ZNF132 staining in BC tissues was performed by two pathologists independently. The staining intensity was defined according to the following criteria: negative, weak, moderate or strong intensity.

### Cell lines and drug treatments

Human breast cancer cell lines MDA-MB-231, MCF7, MDA-MB-453, HCC1937, T47D and DU4475 were used. All cell lines in this study were authenticated by STR analysis in Genesky Co.Ltd. (Shanghai, China) and were excluded the mycoplasma contamination using One-step Quickcolor Mycoplasma Detection Kit (Shanghai Yise Medical Technology Co., Ltd). Cells were cultured in suitable medium with 10% FBS at 37 °C with 5% CO2 concentration. Assumption that 5-aza-2′-deoxycytidine (5-Aza-dC), DNA methyltransferase (DNMT) inhibitor, restores the expression of ZNF132 both mRNA and protein level is yet to be proved. Therefore, the test groups were treated with 5 μM 5-Aza-dC (Sigma-Aldrich) and control groups were treated with the vehicle. When the cell density was up to 80%, RNA was extracted using TRIzol® protocol. qRT-PCR was performed using the Bio-Rad CFX Manager detection system based on the previous description.

### Diagnostic and prognostic significance

Clinic-pathological datas from TCGA dataset were divided into different subgroups according to Age (≥60 /< 60 years), Her2 (Positive/Negative), ER (Positive/Negative), PR (Positive/Negative), Tumor size (T2–4/T1), Lymph node metastasis (Yes/No), Distant metastasis (Yes/No), Clinical stage (II-IV/I) and Expression of ZNF132 (High/Low). Cox proportional hazard regression models were utilized to detect the correlation between these factors and the prognosis of patients with BC. The Kaplan-Meier plotter (http://kmplot.com/) was used to assess the prognosis value of ZNF132 in BC patients including Relapse Free Survival (RFS) and Overall Survival (OS). Furthermore, a reciever operating characteristic curve (ROC) was plotted to determine the ability of ZNF132 expression in distinguishing the difference between BC tissues (*n* = 1104) and adjacent non-tumor tissues (*n* = 114).

### Bioinformatic and DNA methylation analysis

ZNF132 genetic alteration and its impact on the prognosis of BC patients were explored using the cBioPortal OncoPrint (http://www.cBioPortal.org/index.do). Gene Set Enrichment Analysis (http://www.linkedomics.org/) [26] was used to predict potentially biological processes and pathways. In addition, DNA methyltransferases (DNMTs) 1, 3A and 3B and ZNF132 methylation expression including different methylation sites between the BC (*n* = 790) and normal adjacent samples (*n* = 98) from the TCGA database were evaluated to identify the downregulated mechanisms of ZNF132 in BC. Median value of ZNF132 levels was used to divide BC patients (*n* = 1104) into ZNF132^high^ and ZNF132^low^ subgroups. The methylation level of ZNF132 verified by MethHC (http://methhc.mbc.nctu.edu.tw/php/index.php). The relationship between the methylation level of different CpG island sites and gene expression was explored by correlation analysis.

### DNA preparation

Human breast cancer cell lines were routinely cultured in RPMI 1640 or DMEM medium with 10% FBS at 37 °C. When the cell density is about 80%, genomic DNA was extracted from 6 breast cancer cell lines. Briefly, cells were lysed with 1% sodium dodecyl sulfate (SDS) and 0.5 mg/ml proteinase K at 48 °C for 24 h. DNA was subsequently extracted by standard phenol/chloroform protocol, and dissolved in TE buffer, subsequently stored at − 80 °C.

### Sodium bisulfite treatment

A mixture of 4 μg genomic DNA, 10 μg salmon sperm DNA and 0.3 M NaOH was collected. Water was used to top up the final volume to 20 μl. The mixture was incubated at 50 °C for 20 min to denature the DNA. Next, the mixture was transferred into 500 μl of solution containing 3 M sodium bisulfite (Sigma, Saint Louis, MO) and 10 mM hydroquinone (Sigma, Saint Louis, MO) and incubated at 70 °C for 4 h. DNA was then purified using the Wizard DNA Clean-Up System (Promega Corp., Madison, WI). After purification, DNA was followed by ethanol precipitation, dry and dissovled in distilled water.

### Methylation-specific PCR (MSP) assay

Prepare a final 20 μl reaction mixture including 50 ng bisulfite-treated DNA, 16.6 mM ammonium sulfate, 67 mM Tris (pH 8.8), 2 mM MgCl2, 200 μM dNTP, 200 nM primers, and 0.5 U platinum Taq DNA polymerase (Invitrogen Technologies, Inc., CA). The PCR procedure was as follows: 4 min denaturation at 95 °C, then 45 s denaturation at 95 °C, 45 s anneal at 55 °C and 45 s extension at 72 °C. This step was repeated for 35 cycles and finally there was an extension at 72 °C for 5 min. The reaction products were presented in sed on a 1.2% agarose gel and visualized under UV illumination using an ethidium bromide stain along with a positive control and negative control. The primers used are as follow: methylation forward primer: 5′-GTGTAGGGATCGTTATCGC-3′, methylation reverse primer: 5′-AAACGCGTAAC GCTAACTC-3′ and unmethylation forward primer: 5′-TGGGTGTAGGGATTGTTATTGT-3′, unmethylation reverse primer: 5′-CATAACACTAACTCCACTTTCAAA-3′.

### Western blot analysis

Cellular proteins including MCF10A, MDA-MB-231, MCF7, MDA-MB-453, HCC1937, T47D and DU4475 was extracted by the previous description. Anti-ZNF132 antibody (1:2000 dilution) were purchased from BIOSS. Inc. Anti-GAPDH antibody (1:40000 dilution) was purchased from Abgent. Inc. the protein bands were visualized using Western Bright ECL detection system (Advansta, CA).

### DNA methylation and clinical characteristics analysis

The DNA methylation of ZNF132 and clinical characteristics in TCGA was analyzed by MethSurv (https://biit.cs.ut.ee/methsurv/) [27]. The prognostic values and expression levels of CpG methylation in ZNF132 were explored.

### Statistical analysis

All statistical analyses were performed using SPSS 18.0 (IBM Corp., Armonk, NY, USA) and Graphpad Prism 5.0 software. The association between ZNF132 expression and clinical characteristics was analyzed using the Chi-square test. Univariate and multivariate analyses based on the COX regression model were performed to detect the association between clinical variables and the prognosis of BC. Moreover, the ROC curve was used to evaluate the diagnostic capability between BC and adjacent normal tissue. Student’s t-test was used to assess methylation differences of CpG island sites between BC and adjacent normal tissue. *P*-value < 0.05 was considered to indicate a statistically significant difference.

## Results

### Expression of ZNF132 is downregulated in BC

**Fig. 1:**
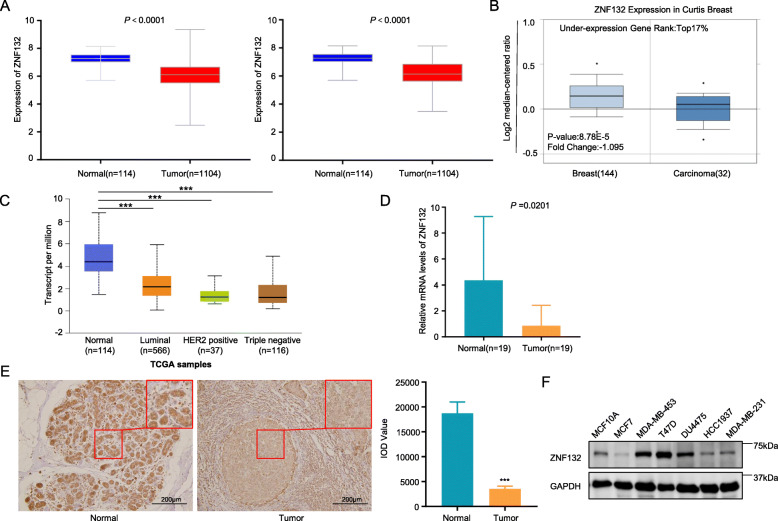
Expression analysis of ZNF132 in BC. **a** ZNF132 was downregulated in 1104 BC tissues and 114 matched pairs of BC tissues compared with adjacent normal tissues based on the TCGA database. **b** Data from the Oncomine 4.5 database also revealed that mRNA expression of ZNF132 was significantly reduced in BC tissues. **c** ZNF132 expression was reduced in different BC molecular subgroups, including luminal A, luminal B, HER2-positive, Triple negative BC. Data were presented as mean ± SD. ***, *P* < 0.001 for comparison with the control. **d** qRT-PCR assay was performed to assess the mRNA expression of ZNF132 in 19 cases of BC tissues. **e** Immunohistochemistry analysis of ZNF132 in BC and adjacent normal tissues (× 20). **f** ZNF132 expression was determined using Western blot analysis in six BC cell lines and normal breast epithelial cells. GAPDH was used as internal control

We first examined mRNA expression of ZNF132 in BC and adjacent noncancerous tissues using TCGA database and Oncomine database. As shown in Fig. 1a and b, ZNF132 was significantly downregulated in BC compared with normal control. The same trend was obtained in different types of breast cancer using UALCAN platform (Fig. 1c). This finding was further supported using the qRT-PCR (Fig. 1d) and immunohistochemical analysis (Fig. 1e). The protein levels of ZNF132 in breast cancer cell lines and normal breast epithelial cell line MCF-10A were examined. The result showed a moderate or slight decrease in MCF7 and HCC1937 cells as compared to MCF-10A. Finally, we attempted to analyze the association of ZNF132 expression with patient clinicopathological features using TCGA dataset. Chi-square test showed that the expression of ZNF132 was significantly associated with HER2 status (*P* = 0.001), ER status (*P* = 0.000), PR status (P = 0.000), tumor size (*P* = 0.006) and lymph node metastasis (*P* = 0.003) (Table 1).

**Table 1:** Clinical association between ZNF132 expression and clinicopathological variables in BC patients

		ZNF132 expression		χ^2^ test *P-* value	Correlation	
Variable	Number	Low	High		*r*	*P*-value
Age						
≤ 60	611	289	321	0.053	−0.046	0.123
>60	493	263	231			
HER2						
Negative	652	292	360	0.001	−0.135	0.000
Positive	114	70	44			
ER						
Negative	179	111	68	0.000	0.209	0.000
Positive	601	266	335			
PR						
Negative	255	157	98	0.000	0.236	0.000
Positive	522	216	306			
Tumor size						
T1	282	121	161	0.006	−0.125	0.000
T2-T4	819	429	390			
Lymph node metastasis						
No	516	420	465	0.003	−0.056	0.064
Yes	568	118	81			
Distant metastasis						
No	964	464	450	0.233	−0.044	0.170
Yes	22	14	8			
Clinical stage						
I	183	80	103	0.058	−0.084	0.006
II--IV	899	462	437			

### ZNF132 was a prognostic factor and diagnostic marker in BC

**Fig. 2:**
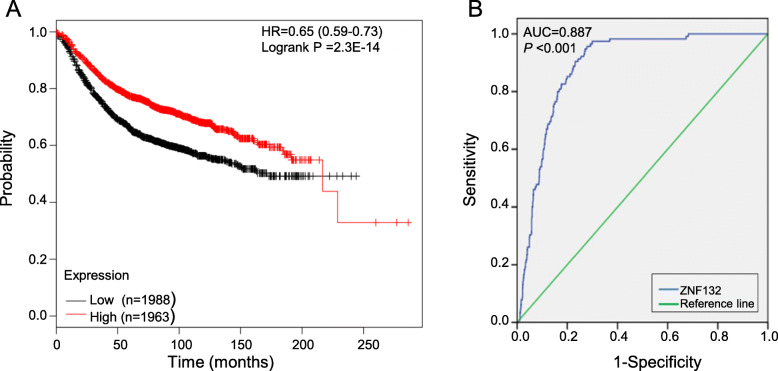
Prognostic and diagnostic value of ZNF132. **a** Survival analysis of ZNF132 in BC showed that low ZNF132 expression was associated with a reduced RFS using Kaplan-Meier Plotter (*n* = 3951). **b** An AUC value of 0.887 revealed a moderate diagnostic value (*P* < 0.001)

The result from the Kaplan-Meier plotter revealed that low ZNF132 expression was significantly associated with a shorter RFS time in BC(Fig. 2,a p=2.3E-14)Univariate COX regression analysis confirmed that some clinical features including age, tumor size, lymph node metastasis, distant metastasis and clinical stage were significantly associated with OS in BC patients (Table2). However, there was no statistical significance between the expression of ZNF132 and the prognosis of BC (HR =1.129, *P* = 0.587) using multivariate analysis (Table3). Moreover, an area under the curve (AUC, representing the accuracy of differentiation) of 0.887 suggested that the level of ZNF132 has sufficient sensitivity and specificity to identify the difference between BC and adjacent normal tissues (Fig. 2b).

### Bioinformatic analysis of ZNF132

**Fig. 3:**
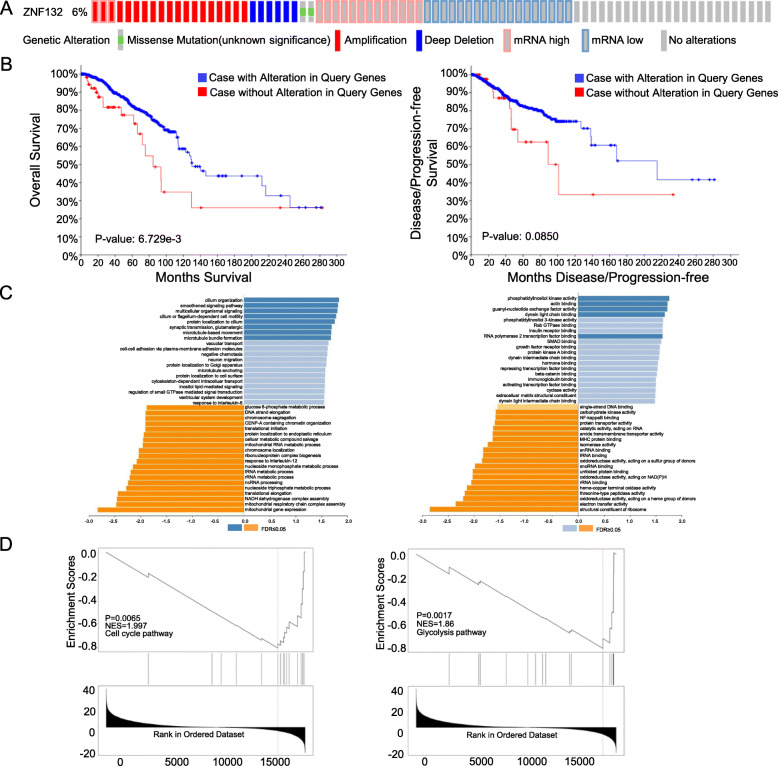
Bioinformatic analysis of ZNF132. **a** A total of 6% (58/960) of GC cases exhibited ZNF132 alteration. **b** OS analysis of BC patients with and without ZNF132 alteration. **c** Potential biological processes and biological pathways in BC were identified by GSEA analysis. **d** ZNF132 expression was negatively correlated with the cell cycle and glycolysis pathway

**Fig. 4:**
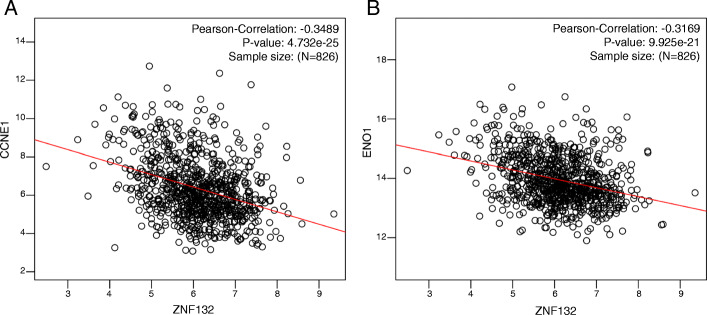
Genes involved in the cell cycle and glycolysis were associated with ZNF132 expression. **a** ZNF132 was negatively correlated with the expression of CCNE1 in cell cycle and (**b**) the expression of ENO1 in glycolysis

The result from cBioPortal revealed that 6% (58/960) of BC exhibited ZNF132 alteration including missense mutation (2/960), amplification (19/960), deep deletion (6/960), mRNA upregulation (13/960) and mRNA downregulation (18/960) (Fig. 3a). Prognosis analysis was performed to explore the influences with and without ZNF132 alteration. The result showed a statistically significant difference existed for OS but not for DFS (Fig. 3b). The analysis from GSEA showed that ZNF132 participated in mediating multiple biological processes including cilium organization, cilium or flagellum-dependent cell motility, synaptic transmission, glutamatergic, microtubule bundle formation, mitochondrial gene expression, mitochondrial respiratory chain complex assembly, ribonucleoprotein complex biogenesis, translational initiation etc. (Fig. 3c). The biological pathways of ZNF132 contained the regulation of cell cycle, glycolysis, cholesterol biosynthesis, ubiquitin proteasome pathway, TCA cycle (Fig. 3d). Cyclin E1 (CCNE1) and Alpha-enolase (ENO1) are located at the core position of the cell cycle and glycolysis regulation gene sets. The correlation analysis indicated that ZNF132 expression was negatively associated with the cyclin E1(Fig. 4a)and ENO1 level (Fig. 4b).

### Hypermethylation of ZNF132 in BC

**Fig. 5:**
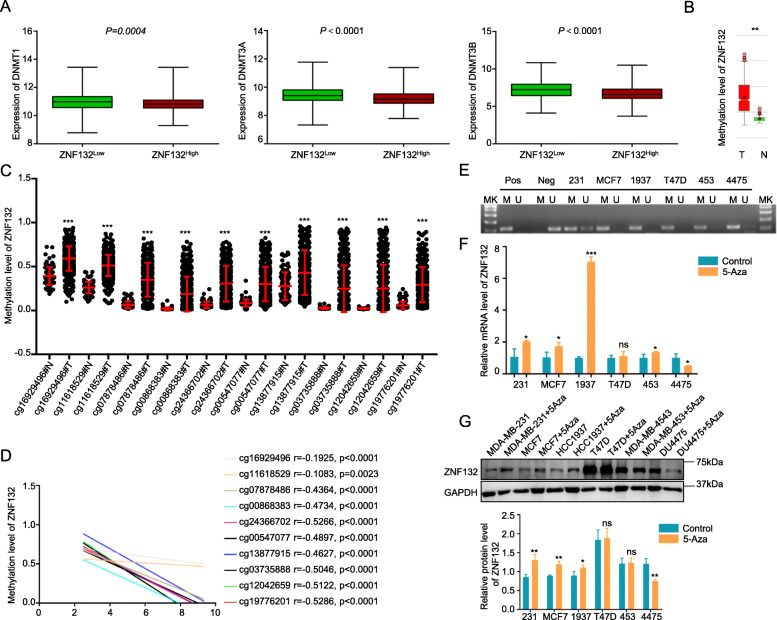
Aberrant methylation of ZNF132 in BC. **a** Expression of 3 DNA methyltransferases (DNMT1, DNMT3A and DNMT3B) in ZNF132^High^ and ZNF132^Low^ group (*n* = 1104). **b** MethHC analysis demonstrated that the methylation level of ZNF132 in BC tissues was significantly higher than the normal sample. **c** Methylation level of ZNF132 in different CpG sites between BC tissues and matched normal tissues. **d** ZNF132 methylation level was negatively correlated with its gene expression. **e** Promoter methylation of ZNF132 in BC cell lines was determined using the MSP assay. In vitro methylated DNA as a positive control for methylated gene (Pos); Bisulfite-modified normal leukocyte DNA as a positive control for unmethylated gene (Neg); Mk, DNA marker; M, methylated gene; U, unmethylated gene; 231, MDA-MB-231; MCF7; 1937, HCC1937; T47D; 453, MDA-MB-453; 4475, DU4475. **f** mRNA level of ZNF132 was partially restored by 5-Aza-dC including MDA-MB-231, MCF7, HCC1937, and MDA-MB-453. **g** The protein level of ZNF132 was partially restored by 5-Aza-dC, including MDA-MB-231, MCF7, and HCC1937. Ctr, Control; 5-Aza, 5-Aza-Dc. Statistically significant differences were indicated: *P < 0.01, **P < 0.001, ***P < 0.0001

Aberrant DNA methylation serves as an important mechanism for downregulation of gene expression and its process involves the cooperative effect among methyltransferases DNMT1, 3A and 3B. Firstly, the expression of DNMT1, DNMT3A and DNMT3B was analyzed in the BC tissues (*n* = 1104) using TCGA dataset. As shown in Fig. 5a, the 3 DNA methyltransferases showed higher expression in the ZNF132^low^ group as compared to ZNF132^high^ group. Result from MethHC [28] (http://methhc.mbc.nctu.edu.tw/php/index.php) also demonstrated that the methylation level of ZNF132 was significantly higher in BC tissues than the normal sample (Fig. 5b). The analysis of different CpG island methylation sites based on the TCGA database also revealed the same trend (Fig. 5c). The ZNF132 methylation level was negatively correlated with its gene expression (Fig. 5d). ZNF132 methylation was detected in a panel of breast cancer cell lines using MSP assay. As shown in Fig. 5f-g 5-Aza-dC could restore the mRNA and protein levels of ZNF132 in partial cell lines including MDA-MB-231, MCF7 and HCC1937 as compared to the untreated normal controls.

### ZNF132 methylation was correlated with prognosis and clinicopathological features of BC

**Fig. 6:**
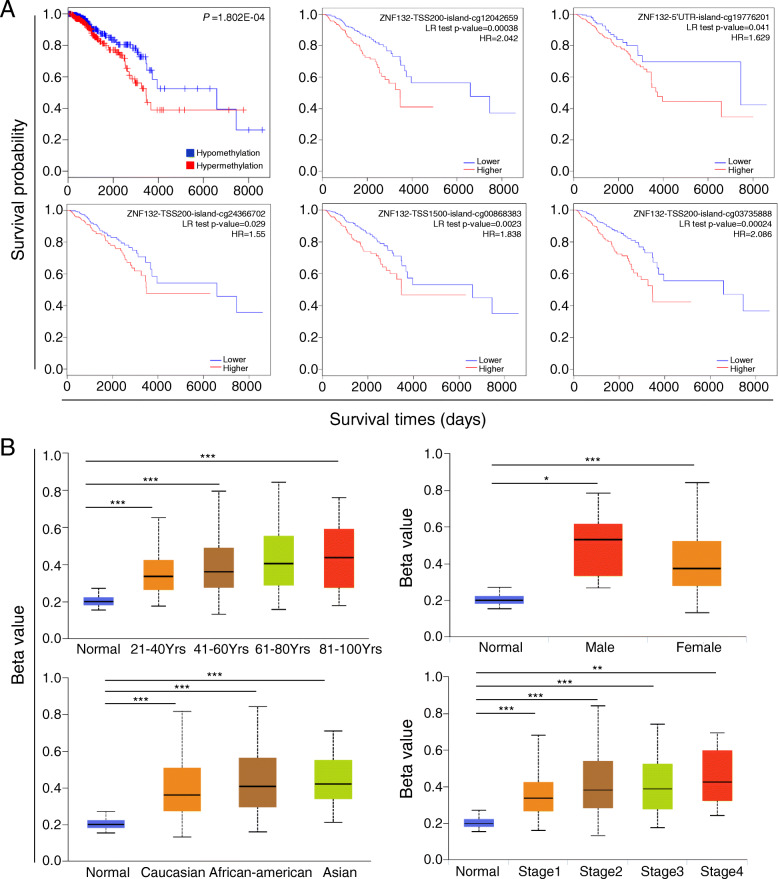
The effect of ZNF132 methylation on prognosis and clinicopathological features. **a** The high methylation level of ZNF132 was negatively correlated with overall survival in BC patients. **b** UALCAN was used to evaluate the impact of aberrant methylation on the clinicopathological features of BC patients

The investigation from MethSurv (https://biit.cs.ut.ee/methsurv/) showed that BC patients with higher ZNF132 methylation had a shorter survival time (Fig. 6a; *P* = 1.802E-04). Survival analyses of different methylated regions also demonstrated a similar trends (Fig. 6a; cg12042695, *P* = 0.00038; cg19776201, *P* = 0.041; cg24366702, *P* = 0.029; cg00868383, *P* = 0.0023; cg03735888, *P* = 0.00024). UALCAN was used to evaluate the impact of aberrant methylation on the clinicopathological features of BC patients. The analysis showed that high methylation levels of ZNF132 were associated with older age and advanced tumor stage in BC patients as compared to low methylation group. The same trend was obtained in Asian regions and male patients (Fig. 6b).

## Discussion

Breast cancer is an aggressive malignant tumor in females. The common metastasis locations are: lungs, bone and brain. BC causes approximately 522,000 deaths yearly [1]. However, the specific mechanism of BC is still unclear. Currently, the causes of BC involve many events including genetics and epigenetics modification. Epigenetic changes include DNA promoter methylation, gene mutation and deletion in tumorigenesis. In the past decades, DNA methylation has been demonstrated to be a promising early diagnostic biomarker for BC. However, useful markers in practice have not been completely identified.

Zinc finger protein is an important family of transcription factors. Majority of human zinc finger proteins contain the KRAB domains. KRAB domains has been proved to act as a transcriptional repressor by interacting with KAP1 and subsequently recruiting histone-modifying proteins [21, 29, 30]. ZNF132, a member of the zinc finger protein family, is only reported to be downregulated by promoter methylation in ECSC and PC [31, 32]. However, its diagnostic and prognostic values have not been elucidated in BC until now. This study is the first one to systematically explore the clinical significance of ZNF132 in BC.

This paper indicates that ZNF132 has a significantly lower expression in BC tissues than adjacent normal tissues both in mRNA and protein level. This implied that ZNF132 can serve as a tumor suppressor in BC. This was in agreement with the study of ZNF132 in ECSC and PC. Larger samples need to be collected to provide more powerful evidence to verify the role of ZNF132 in BC. In addition, the ROC curve revealed that ZNF132 displayed a significant diagnostic value for BC (AUC =0.887, *P* <0.001). Lower ZNF132 expression correlates with worse prognosis of BC. ZNF132 cant be a promising diagnostic and prognostic marker for BC. However, as demonstrated in Table 2 and Table 3, univariate and multivariate analysis failed to provide independent prognostic information for ZNF132 expression. The possible reasons are summarized as follows: a). A series of mixed factors were involved in the prognosis of BC. The common factors were only listed in the present study. b). Samples in this study based on the TCGA database were insufficient to clarify the correlation between ZNF132 expression and prognostic value. Therefore, large-scale prospective study would be necessary to confirm the value of ZNF132 in future. Moreover, analysis from Kaplan-Meier plotter revealed that low ZNF132 expression was associated with a shorter RFS for patients of BC but absent in OS. Therefore, ZNF132 expression can detect BC recurrence. Based on tumor heterogeneous characteristics, BC is classified according to several molecular subgroups: luminal A, luminal B, human epidermal growth factor receptor 2 (HER2), normal and basal-like based on immunohistochemical evaluation of estrogen receptor (ER), progesterone receptor (PR), HER2 and proliferation marker Ki-67. Analysis based on TCGA database revealed that downregulated ZNF132 was correlated with the malignant phenotype of BC. This included positive HER2 status, larger tumor sizes, distant metastasis and advanced clinical stage, indicating ZNF132 suppresses the progression of BC by inhibiting the growth, invasion, and metastasis of tumor cells. ZNF132 expression was positively associated with ER and PR status. Further investigation is needed to determine whether combined detection of ZNF132 together with other molecules would be valuable in improving prognosis assessment.

**Table 2:** Univariate analysis of prognostic factors of BC

Variable	Hazard ratio	95%CI	*P*-value
Age(>60/≤60)	1.928	(1.402,2.652)	0.000
HER2(Positive / Negative)	1.402	(0.592,1.834)	0.886
ER(Positive/Negative)	1.018	(0.659,1.573)	0.937
PR(Positive/Negative)	0.938	(0.637,1.379)	0.744
Tumor size(T2-T4/T1)	1.500	(1.020,2.206)	0.040
Lymph Node metastasis(Yes/No)	2.200	(1.542,3.140)	0.000
Distant metastasis(Yes/No)	4.749	(2.840,7.940)	0.000
Clinical stage(II-IV/I)	2.249	(1.337,3.783)	0.002
ZNF132 expression(High/Low)	0.829	(0.602,1.143)	0.252

**Table 3:** Multivariate analysis of prognostic factors of BC

Variable	Hazard ratio	95%CI	*P*-value
Age(>60/≤60)	2.278	(1.453,3.571)	0.000
HER2(Positive / Negative)	0.804	(0.405,1.597)	0.533
ER(Positive/Negative)	1.015	(0.507,2.032)	0.966
PR(Positive/Negative)	0.618	(0.330,1.154)	0.131
Tumor size(T2-T4/T1)	1.238	(0.548,2.800)	0.608
Lymph Node metastasis(Yes/No)	1.630	(0.959,2.770)	0.071
Distant metastasis(Yes/No)	3.427	(1.665,7.055)	0.001
Clinical stage(II-IV/I)	1.140	(0.386,3.369)	0.813
ZNF132 expression(High/Low)	1.129	(0.728,1.753)	0.587

The first investigation from cBioPortal showed that approximately 6% BC patients exhibited ZNF132 alterations. The mRNA downregulation was the predominant type of alteration. This could contribute to the downregulation of ZNF132 in BC. This helped recognize the potential mechanisms of ZNF132 in BC. In addition, the analysis based on GSEA demonstrated that ZNF132 participated in a variety of important biological processes and pathways. CCNE1 and ENO1 are located at an important position in these biological processes. Cyclin E1 (CCNE1) belongs to the highly conserved cyclin family and forms a complex with CDK2 whose activity is required for cell cycle G1/S transition. CCNE1 has been reported to upregulated in various human cancer including breast [33], bladder [34] and ovarian [35]. CCNE1 mediates premature S-phase entry, ineffective DNA replication and genomic instability. Alpha-enolase (ENO1) is a prominent glycolytic enzyme. ENO1 was upregulated in multiple cancers and its overexpression was involved in tumor cell proliferation and metastasis such as glioma [36], gastric [37], pancreatic [38], colorectal [39], BC [40] etc. ZNF132 expression was negatively associated with CCNE1 and ENO1 level based on the correlation analysis. Therefore ZNF132 might inhibit the progression of BC by regulating the expression of ENO1 and CCNE1.

Aberrant promoter methylation permanently inactivate tumor-associated genes, particularly tumor suppressor genes [41]. In this study, ZNF132 methylation level was higher in BC tissue than that in normal tissues. Moreover, 3 DNA methyltransferases were overexpressed in the ZNF132^low^ group. Methylated modification of ZNF132 was detected in 6 BC cell lines. 5-Aza-dC treatment restores the mRNA and protein levels of ZNF132 in some cell lines including MDA-MB-231, MCF7 and HCC1937. However, 5-Aza-dC failed to restore the expression of ZNF132 between the mRNA and protein levels in the T47D cell. Besides, the MDA-MB-453 cell failed to restore ZNF132 expression at the protein level. Moreover, an opposite trend was obtained in DU4475. The most likely reason is that the ZNF132 gene may undergo other epigenetic modifications including transcription and post-transcriptional regulation. Finally, methylation analysis in 10 CpG island sites including cg169294963, cg11618529, cg07878486, cg00868383, cg24366702, cg00547077, cg13877915, cg03735888, cg12042659 and cg19776201 indicated that these sites were hypermethylated in BC sample. This suggested that DNA methylation in these sites inactivates ZNF132 gene transcription. Clinical samples analysis provided strong evidence between promoter methylation status and expression of ZNF132 in BC. Therefore, ZNF132 hypermethylation act as an independent risk factor in BC patients.

## Conclusion

In conclusion, this research demonstrated the expression, diagnostic ability and prognostic significance of ZNF132 based on the TCGA database. Aberrant hypermethylation of ZNF132 mediated its silence in BC. Therefore, ZNF132 could be used as a potential target for diagnosis and prognostic evaluation in BC.

## Supplementary Information

**Additional file 1: Supplement Fig.5E.** Original gel image.

**Additional file 2: Supplement Fig.1F** & **Fig.5G.** Original blots image.

## Data Availability

The datasets used and analyzed during the current study are available from TCGA database (http://cancergenome.nih.gov/) and multiple online tools, including Oncomine™ database (www.oncomine.org), UALCAN platform (http://ualcan.path. uab.edu/) and Xena platform (https://xenabrowser.net/).

## Abbreviations

TCGA: The Cancer Genome Atlas
ZNF132: Zinc Finger Protein 132
AUC: An area under the curve
ROC: Receiver operating characteristic curve
OS: Overall survival
RFS: Relapse-free survival
DFS: Disease-free survival
BC: Breast cancer
GSEA: Gene Set Enrichment Analysis
MSP: Methylation-specific PCR

## References

[1] Ferlay J, Soerjomataram I, Dikshit R, Eser S, Mathers C, Rebelo M, Parkin DM, Forman D, Bray F (2015). Cancer incidence and mortality worldwide: sources, methods and major patterns in GLOBOCAN 2012. Int J Cancer.

[2] DeSantis C, Ma J, Bryan L, Jemal A (2014). Breast cancer statistics, 2013. CA Cancer J Clin.

[3] Kolacinska A, Herman K, Morawiec J, Paszek S, Zawlik I, Sliwczynski A (2019). Improvement in outcomes of breast cancer patient treatment in Poland in the 21st century. Breast J.

[4] Koroukian SM, Bakaki PM, Htoo PT, Han X, Schluchter M, Owusu C, Cooper GS, Rose J, Flocke SA (2017). The breast and cervical Cancer early detection program, Medicaid, and breast cancer outcomes among Ohio's underserved women. Cancer.

[5] Howard DH, Tangka FK, Royalty J, Dalzell LP, Miller J, O'Hara B, Joseph K, Kenney K, Guy G, Hall IJ (2015). Breast cancer screening of underserved women in the USA: results from the National Breast and cervical Cancer early detection program, 1998-2012. Cancer Causes Control : CCC.

[6] Miller JW, Hanson V, Johnson GD, Royalty JE, Richardson LC (2014). From cancer screening to treatment: service delivery and referral in the National Breast and cervical Cancer early detection program. Cancer.

[7] Miller JW, Plescia M, Ekwueme DU (2014). Public health national approach to reducing breast and cervical cancer disparities. Cancer.

[8] Plescia M, Wong F, Pieters J, Joseph D (2014). The National Breast and cervical Cancer early detection program in the era of health reform: a vision forward. Cancer.

[9] Siegel RL, Miller KD, Jemal A (2018). Cancer statistics, 2018. CA Cancer J Clin.

[10] Howlader NNAKM, Neyman N, Aminou R, Waldron W, Altekruse SF, Kosary CL, Ruhl J, Tatalovich Z, Cho H, Mariotto A, Eisner MP, Lewis DR, Chen HS, Feuer EJ, Cronin KA, Edwards BK (2011). SEER cancer statistics review, 1975–2008.

[11] Gyorffy B, Bottai G, Fleischer T, Munkacsy G, Budczies J, Paladini L, Borresen-Dale AL, Kristensen VN, Santarpia L (2016). Aberrant DNA methylation impacts gene expression and prognosis in breast cancer subtypes. Int J Cancer.

[12] Jovanovic J, Ronneberg JA, Tost J, Kristensen V (2010). The epigenetics of breast cancer. Mol Oncol.

[13] Polyak K (2007). Breast cancer: origins and evolution. J Clin Invest.

[14] Rodriguez-Paredes M, Esteller M (2011). Cancer epigenetics reaches mainstream oncology. Nat Med.

[15] Tapia T, Smalley SV, Kohen P, Munoz A, Solis LM, Corvalan A, Faundez P, Devoto L, Camus M, Alvarez M (2008). Promoter hypermethylation of BRCA1 correlates with absence of expression in hereditary breast cancer tumors. Epigenetics.

[16] Esteller M, Corn PG, Baylin SB, Herman JG (2001). A gene hypermethylation profile of human cancer. Cancer Res.

[17] Widschwendter M, Jones PA (2002). DNA methylation and breast carcinogenesis. Oncogene.

[18] Wittenberger T, Sleigh S, Reisel D, Zikan M, Wahl B, Alunni-Fabbroni M, Jones A, Evans I, Koch J, Paprotka T, Lempiäinen H, Rujan T, Rack B, Cibula D, Widschwendter M (2014). DNA methylation markers for early detection of women's cancer: promise and challenges. Epigenomics.

[19] Raffel S, Falcone M, Kneisel N, Hansson J, Wang W, Lutz C, Bullinger L, Poschet G, Nonnenmacher Y, Barnert A, Bahr C, Zeisberger P, Przybylla A, Sohn M, Tönjes M, Erez A, Adler L, Jensen P, Scholl C, Fröhling S, Cocciardi S, Wuchter P, Thiede C, Flörcken A, Westermann J, Ehninger G, Lichter P, Hiller K, Hell R, Herrmann C, Ho AD, Krijgsveld J, Radlwimmer B, Trumpp A (2017). BCAT1 restricts αKG levels in AML stem cells leading to IDHmut-like DNA hypermethylation. Nature.

[20] Jen J, Wang YC (2016). Zinc finger proteins in cancer progression. J Biomed Sci.

[21] Fedotova AA, Bonchuk AN, Mogila VA, Georgiev PG (2017). C2H2 zinc finger proteins: the largest but poorly explored family of higher eukaryotic transcription factors. Acta Nat.

[22] Tomczak K, Czerwińska P, Wiznerowicz M. The Cancer Genome Atlas (TCGA): an immeasurable source of knowledge. *Contemporary oncology (Poznan, Poland)*, 2015. **19**(1a):A68–77. 10.5114/wo.2014.47136 PMC4322527 25691825

[23] Rhodes DR, Yu J, Shanker K, Deshpande N, Varambally R, Ghosh D, Barrette T, Pandey A, Chinnaiyan AM (2004). ONCOMINE: a cancer microarray database and integrated data-mining platform. Neoplasia (New York, NY).

[24] Chandrashekar DS, Bashel B, Balasubramanya SAH, Creighton CJ, Ponce-Rodriguez I, Chakravarthi B, Varambally S (2017). UALCAN: A Portal for Facilitating Tumor Subgroup Gene Expression and Survival Analyses. Neoplasia (New York, NY).

[25] Rio DC, Ares M, Hannon GJ, Nilsen TW (2010). Purification of RNA using TRIzol (TRI reagent). Cold Spring Harbor protocols.

[26] Vasaikar SV, Straub P, Wang J, Zhang B (2018). LinkedOmics: analyzing multi-omics data within and across 32 cancer types. Nucleic Acids Res.

[27] Modhukur V, Iljasenko T, Metsalu T, Lokk K, Laisk-Podar T, Vilo J (2018). MethSurv: a web tool to perform multivariable survival analysis using DNA methylation data. Epigenomics.

[28] Huang WY, Hsu SD, Huang HY, Sun YM, Chou CH, Weng SL, Huang HD (2015). MethHC: a database of DNA methylation and gene expression in human cancer. Nucleic Acids Res.

[29] Margolin JF, Friedman JR, Meyer WK, Vissing H, Thiesen HJ, Rauscher FJ (1994). Krüppel-associated boxes are potent transcriptional repression domains. Proc Natl Acad Sci U S A.

[30] Vissing H, Meyer WK, Aagaard L, Tommerup N, Thiesen HJ (1995). Repression of transcriptional activity by heterologous KRAB domains present in zinc finger proteins. FEBS Lett.

[31] Jiang D, He Z, Wang C, Zhou Y, Li F, Pu W, Zhang X, Feng X, Zhang M, Yecheng X, Xu Y, Jin L, Guo S, Wang J, Wang M (2018). Epigenetic silencing of ZNF132 mediated by methylation-sensitive Sp1 binding promotes cancer progression in esophageal squamous cell carcinoma. Cell Death Dis.

[32] Abildgaard MO, Borre M, Mortensen MM, Ulhoi BP, Torring N, Wild P, Kristensen H, Mansilla F, Ottosen PD, Dyrskjot L (2012). Downregulation of zinc finger protein 132 in prostate cancer is associated with aberrant promoter hypermethylation and poor prognosis. Int J Cancer.

[33] Zhao ZM, Yost SE, Hutchinson KE, Li SM, Yuan YC, Noorbakhsh J, Liu Z, Warden C, Johnson RM, Wu X, Chuang JH, Yuan Y (2019). CCNE1 amplification is associated with poor prognosis in patients with triple negative breast cancer. BMC Cancer.

[34] Matsushita R, Seki N, Chiyomaru T, Inoguchi S, Ishihara T, Goto Y, Nishikawa R, Mataki H, Tatarano S, Itesako T, Nakagawa M, Enokida H (2015). Tumour-suppressive microRNA-144-5p directly targets CCNE1/2 as potential prognostic markers in bladder cancer. Br J Cancer.

[35] Nakayama N, Nakayama K, Shamima Y, Ishikawa M, Katagiri A, Iida K, Miyazaki K (2010). Gene amplification CCNE1 is related to poor survival and potential therapeutic target in ovarian cancer. Cancer.

[36] Song Y, Luo Q, Long H, Hu Z, Que T, Zhang X, Li Z, Wang G, Yi L, Liu Z (2014). Alpha-enolase as a potential cancer prognostic marker promotes cell growth, migration, and invasion in glioma. Mol Cancer.

[37] Sun L, Lu T, Tian K, Zhou D, Yuan J, Wang X, Zhu Z, Wan D, Yao Y, Zhu X, He S (2019). Alpha-enolase promotes gastric cancer cell proliferation and metastasis via regulating AKT signaling pathway. Eur J Pharmacol.

[38] Shen J, Person MD, Zhu J, Abbruzzese JL, Li D (2004). Protein expression profiles in pancreatic adenocarcinoma compared with normal pancreatic tissue and tissue affected by pancreatitis as detected by two-dimensional gel electrophoresis and mass spectrometry. Cancer Res.

[39] Zhan P, Zhao S, Yan H, Yin C, Xiao Y, Wang Y, Ni R, Chen W, Wei G, Zhang P (2017). Alpha-enolase promotes tumorigenesis and metastasis via regulating AMPK/mTOR pathway in colorectal cancer. Mol Carcinog.

[40] Tu SH, Chang CC, Chen CS, Tam KW, Wang YJ, Lee CH, Lin HW, Cheng TC, Huang CS, Chu JS, Shih NY, Chen LC, Leu SJ, Ho YS, Wu CH (2010). Increased expression of enolase alpha in human breast cancer confers tamoxifen resistance in human breast cancer cells. Breast Cancer Res Treat.

[41] Shi J, Liu W, Sui F, Lu R, He Q, Yang Q, Lv H, Shi B, Hou P (2015). Frequent amplification of AIB1, a critical oncogene modulating major signaling pathways, is associated with poor survival in gastric cancer. Oncotarget.

