# Supplementary figures and images for: Downregulated ZNF132 predicts unfavorable outcomes in breast Cancer via Hypermethylation modification
Source: BMC Cancer. 2021 Apr 7;21:367. doi: 10.1186/s12885-021-08112-z (PMC8028803; doi:10.1186/s12885-021-08112-z)

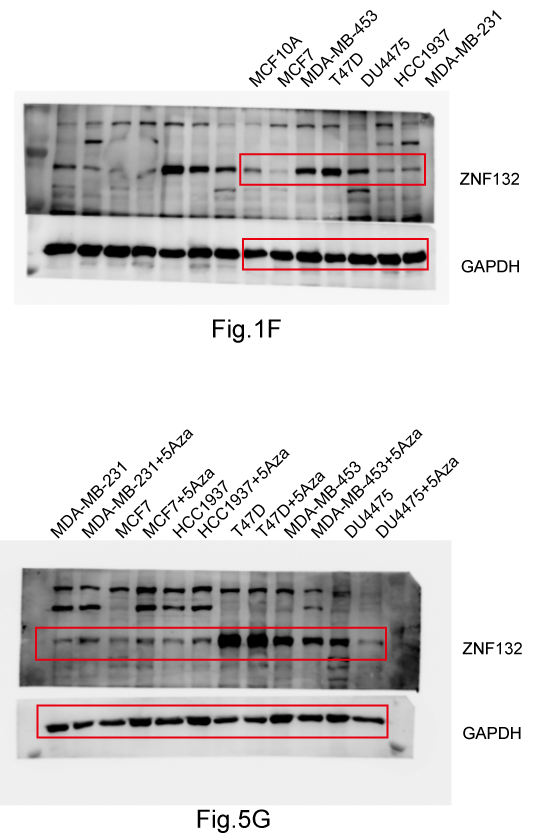

Supplement: Supplementary file 1 — Additional file 1: Supplement Fig.5E. Original gel image. [file 12885_2021_8112_MOESM1_ESM.tif]

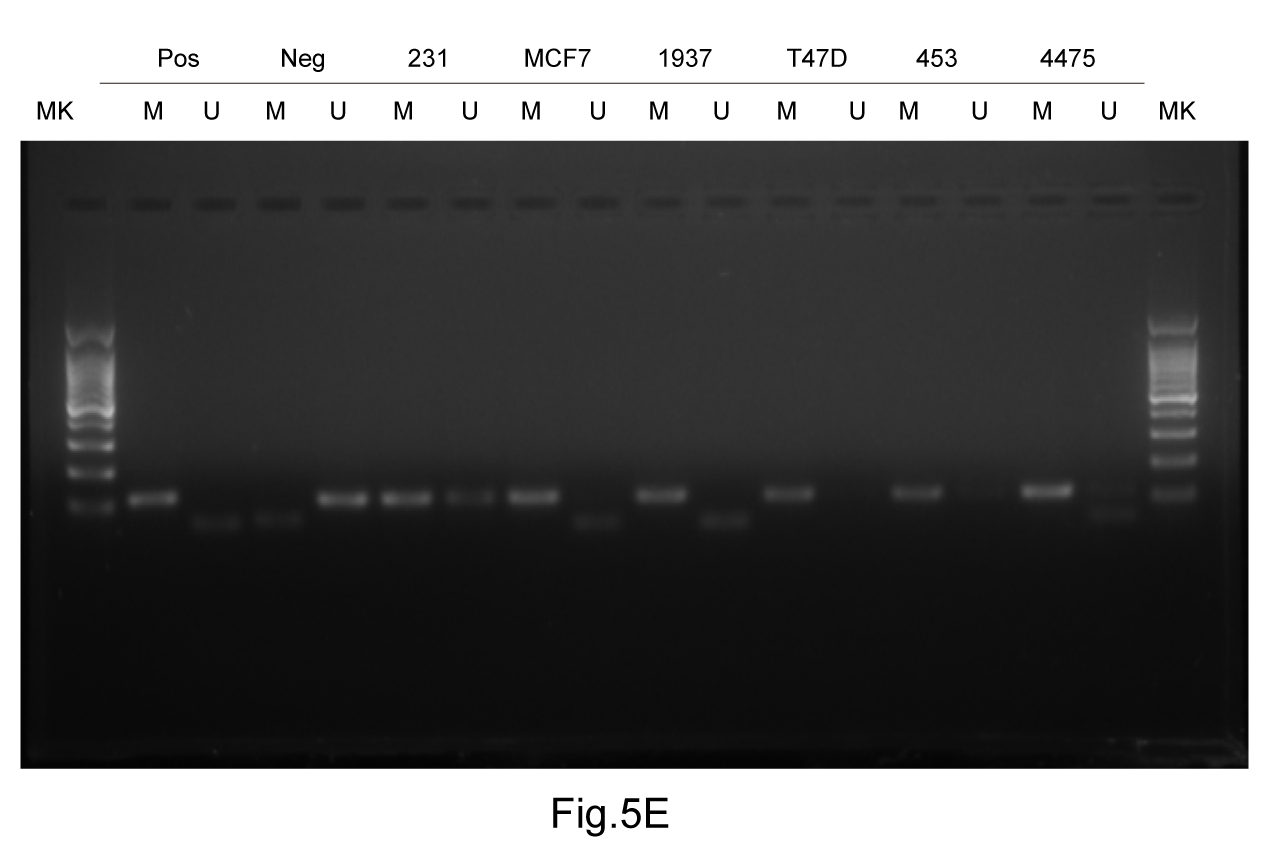

Supplement: Supplementary file 2 — Additional file 2: Supplement Fig.1F & Fig.5G. Original blots image. [file 12885_2021_8112_MOESM2_ESM.tif]
